# Recurrent lipoma: an uncommon presentation in the wrist after incomplete excision

**DOI:** 10.1080/23320885.2024.2303997

**Published:** 2024-01-18

**Authors:** Marc D. Mazur, Tinatin Natroshvili, Sandra J.M. Jongen, Marius A. Kemler

**Affiliations:** Department of Plastic Surgery, Martini Hospital Groningen

**Keywords:** Lipoma, reoperation, guyon canal, carpal tunnel, recurrence, hand, surgery, hand

## Abstract

**Patient:**

Female, 58-year-old

**Final Diagnosis:**

Benign recurrent lipoma following incomplete surgical removal.

**Symptoms:**

Discomfort, Aesthetic Dissatisfaction

**Clinical Procedure:**

Surgical Revision-Excision—Exploration with Lipoma Extraction

**Specialty:**

Plastic Surgery (Hand Surgery)

**Objective:**

Unusual Clinical Presentation and Course

**Background:**

Lipoma is a usually painless tumor composed of adipocytes, of fat cells, arising from mesenchymal tissue. It manifests itself in locations in the body where adipocytes are and has circumscribed growth. Its incidence in the hand is relatively low (1%−4.9%). Despite most lipomas being benign and usually asymptomatic, the location of lipoma can lead to nerve compression symptoms. We report a case of an unusual recurrence of lipoma in the wrist after incomplete excision.

**Case report:**

A 58-year-old female presented with a large, soft mass located on the volar side of the wrist, which recurred during the first week following the initial excision. While the patient did not exhibit symptoms of nerve compression, she reported experiencing swelling and pain at the surgical site postoperatively. The patient underwent surgical re-excision of the lesion, and the excised tissue was sent for histological examination. The subsequent histological analysis confirmed the diagnosis of a benign lipoma. The patient expressed satisfaction with the surgical revision, postoperative care, and outcomes, reporting high levels of contentment in pain relief, functional improvement, and cosmetic results.

**Conclusions:**

Lipomas often remain asymptomatic for extended periods, only becoming a source of discomfort or concern once they increase in size or impact one’s appearance. Although most lipomas are benign and pose little risk to overall health, certain malignant variants exist. Recurrence of lipoma is uncommon and typically suggests an incomplete initial excision. In anatomically complex regions like the hand or wrist, meticulous planning and preoperative imaging are essential to prevent compression, exclude malignancy, and preserve function.

## Introduction

Lipomas are soft encapsulated masses of mature adipocytes, easily separated from surrounding tissue. They can arise anywhere in the body where fat cells are but present most commonly on the torso. Lipomas can be found at varying depths within tissues: superficial lipomas are confined to the subcutaneous fatty tissue, deep-seated lipomas grow under the enclosing fascia, and intramuscular lipomas – which are less common – arise within muscles [1]. All these subclassifications are always capsulated and can present as sessile, pedunculated, or submerged growths. Lipomas commonly occur as singular lesions and vary in size from small (2 cm, usually well-rounded) to large (10 cm, often poorly defined and lobular) masses. When multiple lesions are present, it may indicate an underlying syndrome such as neurofibromatosis, Gardner’s syndrome, Dercum’s disease, familial multiple lipomatosis, Proteus syndrome, or Cowden syndrome [2]. The differential diagnosis of lipomas usually includes sebaceous (epidermoid) cysts, hematomas, abscesses, panniculitis, and other malignant forms of adipocytic tumors such as liposarcoma. While lipoma itself is quite frequent, occurrence in the hand is relatively rare and accounts for only 1% to 4.9% of tumors in this anatomical region or 1–5 out of 100 people [3–5]. In general, lipomas are considered a painless benign soft-tissue tumor, and they can exist for years without patients noticing. However, when they grow near nerves their size can cause compression and they can become symptomatic. Additionally, malignant counterparts to the lipoma exist – namely liposarcoma which presents with low levels of differentiation rather than with mature adipocytes [6]. Moreover, intermediate forms have also been described which are referred to as atypical lipomatous tumors/well-differentiated liposarcoma (ALT/WDLPS), which do not metastasize, but can dedifferentiate over time and become malignant [6]. In 2013, the World Health Organization created a Classification of Soft Tissue Tumors, providing an overview of adipocytic tumors and organizing them into 11 benign subtypes, one intermediate category, and five categories of adipose malignancies [7]. The malignant progression of lipomas remains uncertain, but larger lipomas are viewed as having an increased risk of malignancy [7,8]. Recurrence of lipomas is also rare with reports showing recurrence rate of around 5%, usually citing incomplete surgical removal as the cause [9]. Recurrence is also a potential sign of underlying malignancy or dedifferentiation. In this case report, we describe a case of a 58-year-old healthy female with a recurrence of lipoma at the volar aspect of the left wrist within 5 days after the initial surgery.

## Case report

**Figure 1.**
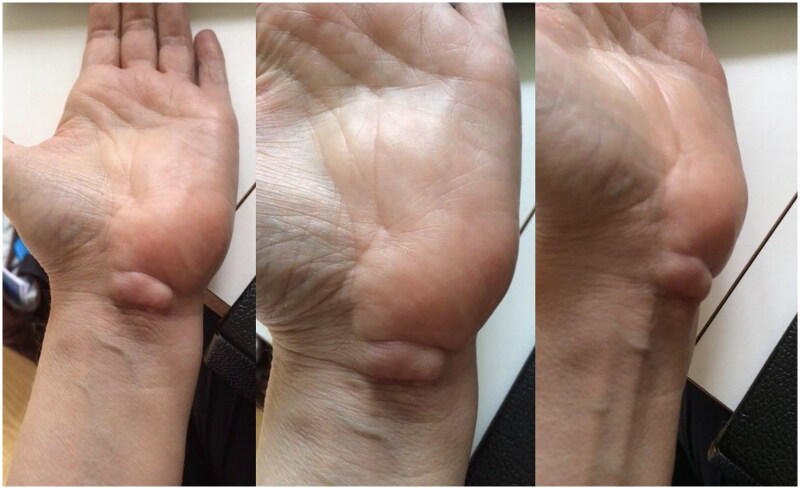
Recurrent lipoma as presented during physical examination.

**Figure 2.**
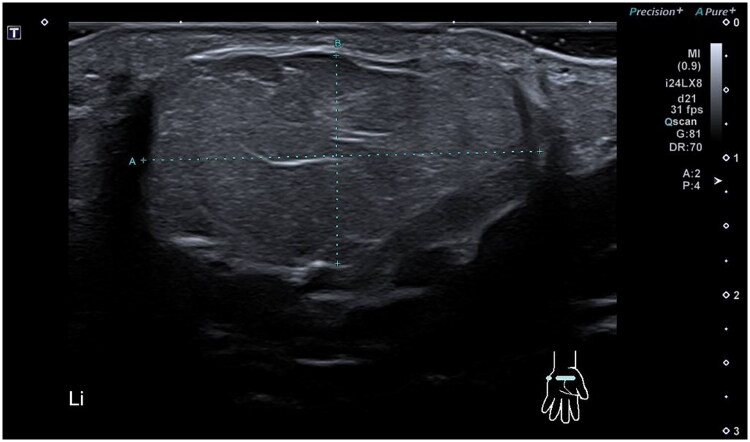
Ultrasound cross-section of the lipoma.

**Figure 3.**
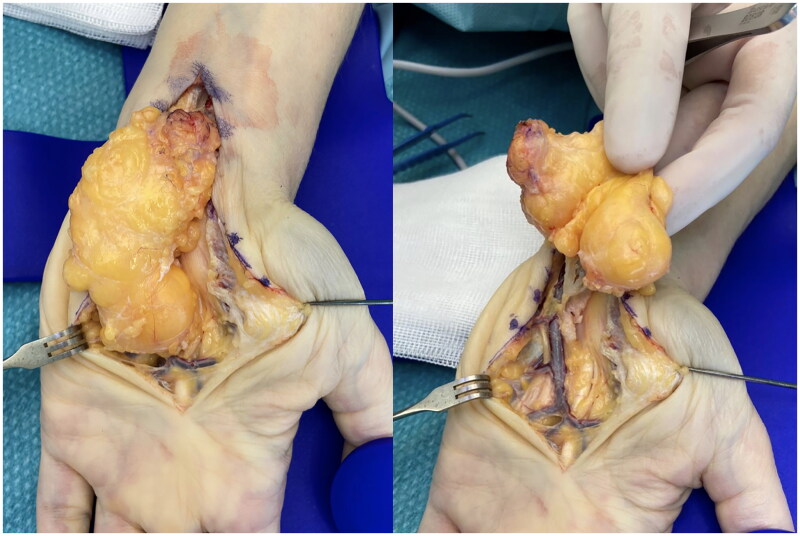
Intra-operative photographs of lipoma recurrence.

**Figure 4.**
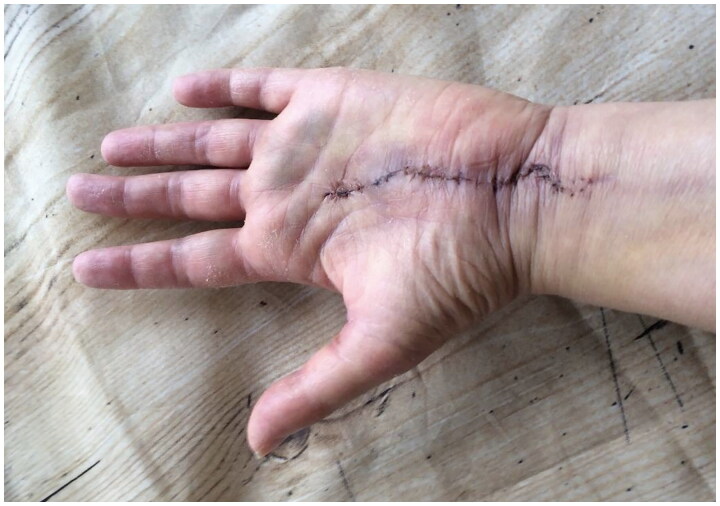
Two weeks post-operative showing the chosen incision.

**Figure 5.**
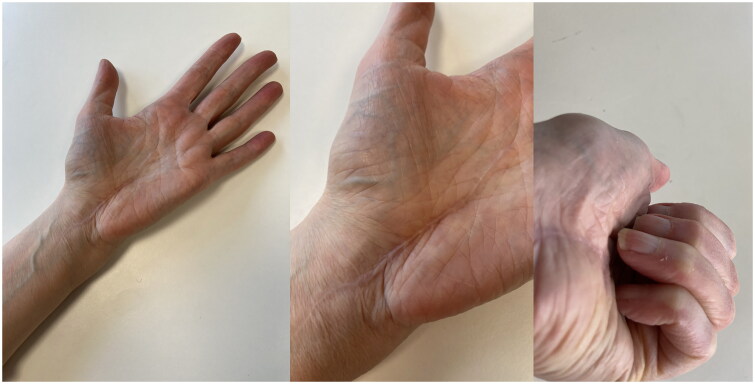
Status 6 months post-op with silicone treatment of scar-tissue. (right) Slight limitation of end range of motion of flexion of the fourth digit.

In May 2021, a 58-year-old, otherwise healthy right-handed female presented to our department with a recurrence of a lipoma on the volar side of her left wrist. The patient reported the recurrence five days after the initial surgery, performed at a different hospital in March 2021, and noticed continued growth until admission. The patient’s chief complaints were pain, decreased function, and discomfort during daily activities. Physical examination revealed a palpable mass with a diameter of approximately 5 cm extending from the wrist to the base of the left hypothenar eminence (Figure 1). No signs of median or ulnar nerve compression were observed upon examination. The patient had no history of trauma, congenital malformation, or other diseases. Ultrasound examination in June 2021 at our clinic revealed a homogenous mass measuring about 6 × 3 × 1.5 cm without increased vascularization, iso-echoic to the surrounding subcutaneous fatty tissue (Figure 2). The radiologist suggested that it was most likely a lipoma, but its location made imaging somewhat difficult, and an MRI was advised as well. Because histological examination of the removed tissue from the original surgery revealed a lipoma, an MRI was deemed unnecessary, and the patient was scheduled for re-excision of the lipoma in her wrist. In November 2021, a revision excision was performed on the patient. As this was a revision surgery involving exploration, the re-excision procedure utilized blockade of the brachial plexus with sedation to ensure the patient’s comfort. The surgical approach involved a new incision was made in along the length of the wrist, as in opening both the carpal tunnel surgeries, and proceeded diagonally towards Guyon’s canal to remove the lipoma to ensure a comprehensive view of the lipoma for proper extraction (Figure 3). During the operation, it was found revealed that the lipoma was larger than expected anticipated based on imaging and had grown into both the carpal tunnel and Guyon’s canal. The ingrowths into and between the carpal tunnel and Guyon’s canal were excised, and any remaining strands were also removed. The lipoma extended until just proximal to the superficial palmar arc. Intraoperatively, the median nerve appeared more fatty than normal. Histology confirmed that the mass – which weighed around 19 g and was 5 × 3.5 × 1.5 cm – was removed *in toto* with the associated capsule (Table 1). After the surgery, the patient was referred to a hand therapist and had a follow-up with the operator six weeks post-operatively. Postoperative outcomes of the lipoma excision and revision surgery include assessments of pain levels, hand and wrist function, patient-reported quality of life, scar appearance, neurological status, and documentation of any complications. At the initial follow-up, the patient had diminished flexion of the fingers and dorsiflexion of the wrist due to scar tissue from the excision and post-operative swelling and immobilization (Figure 4). Physical therapy check-ups were initially performed every other week and then monthly to improve wrist strength, flexibility, and flexion of the fingers. At six months post-op, the patient had achieved 20 degrees wrist extension with the help of silicone treatment of the scar tissue (Figure 5). With exercises, the patient was able to achieve full flexion of all digits except for fourth digit. During the last postoperative visit with the hand therapist, the patient conveyed overall contentment with the surgical revision, postoperative care, and outcomes, reporting high satisfaction levels in pain relief, functional improvement, and cosmetic results, with a minor concern specifically related to limited range of motion of the fourth digit that did not impact or limit daily activities. The patient successfully completed physical therapy and regained normal strength and range of motion without limitations of daily activities. However, a slight limitation of the end range of motion of flexion of fourth digit was noted, which was not present prior to the operative treatment.

**Table 1.** Table of histology reports from both hospitals where surgeries occurred.

Histology reports		
**Location**	Assen	Groningen
**Date**	March 2021	November 2021
**Macro**	A fragmented fat sample with a maximum diameter of 3 cm and a weight of 2 g.	A disc-shaped fat sample measuring 5 × 3.5 × 1.5 cm with membrane. The sample weight is 19 g.
**Micro**	Lobed with thin fibrovascular septa. The adipocytes are of equal size with small hyper chromatic nuclei. No lipoblasts or mitosis.	Sections through tissue fragment - taken up by mature adipose tissue made up of regularly shaped fat cells. Some inflammatory cells including foam cell macrophages are present. No malignant features.
**Conclusion**	Excision of swelling left wrist, volar: Consistent with lipoma.	Soft tissue excision left wrist/palm: lipoma.

## Discussion

Lipomas in the wrist are relatively uncommon, comprising less than 5% of tumors in this anatomical region [2–4]. While malignant tumors in the wrist are rare, their clinical presentation can mimic that of a benign lesion, necessitating careful evaluation to avoid missing potential malignancies. Recurrence of lipomas, although primarily attributed to incomplete surgical removal, can also be an indication of malignancy. Histopathological examination plays a definitive role in confirming the diagnosis of lipoma and ruling out other adipocytic tumors.

In this case, histological analysis of the excised tissue confirmed the diagnosis of a benign lipoma. The examination revealed characteristic features of lipomas, including mature adipocytes with a well-defined capsule. These findings corroborated the initial diagnosis and provided reassurance regarding the benign nature of the lesion. Histopathology remains a crucial component of the diagnostic workup, aiding in distinguishing lipomas from other adipocytic tumors and guiding appropriate management decisions.

Additionally, there were no symptoms of median nerve compression reported by the patient. However, given the slow-growing nature of these lesions, it is anticipated that with time, the lipoma may reach a size that causes nerve compression symptoms. Although median nerve compression is infrequent, even in large lipomas, isolated cases have been previously documented [9,10]. Furthermore, the location of a lipoma in the hand or wrist can potentially impede fine motor skills and lead to a sense of clumsiness in the affected individual. While no median nerve compression symptoms were reported in this case, the realistic risk of the development of compression over time should be acknowledged. Therefore, when encountering a palpable mass with or without compression symptoms in the wrist, it is advisable for the surgeon to consider ruling out any underlying anatomical anomalies associated with variations in osseous, muscular, nerve, or vascular structures [11].

The surgical approach and techniques used in the re-excision of the lipoma in the wrist play a crucial role in achieving complete excision and preventing recurrence. In this case, a revision excision was performed using an incision like that of carpal tunnel surgeries, extending diagonally towards Guyon’s canal. This approach allowed adequate exposure of the lipoma and surrounding structures, facilitating meticulous dissection and removal of the lipoma mass. Notably, intraoperative findings revealed that the lipoma was larger than anticipated, with extensions into the carpal tunnel and between the carpal tunnel and Guyon’s canal. Careful excision of these ingrowths was performed, ensuring complete removal. The surgical technique employed in this case highlights the importance of meticulous planning, extensive dissection, and attention to anatomical variation in complex regions like the wrist.

However, post-operative difficulty primarily arose from the presence of the incision scar, highlighting the significance of scar management techniques in optimizing patient outcomes. Postoperative functional outcomes and rehabilitation are vital considerations in the management of lipomas in the wrist. In this case, the patient experienced diminished flexion of the fingers and dorsiflexion of the wrist postoperatively due to scar tissue formation and swelling. Physical therapy played a pivotal role in the patient’s recovery, frequent check-ups focused on improving wrist strength, flexibility, and finger flexion. At 6 months post-op, the patient achieved significant improvement, with a near-normal range of motion and normal strength in the affected wrist and hand. These outcomes underscore the importance of tailored hand therapy interventions, consistent follow-ups, and patient compliance in achieving optimal functional recovery.

## Conclusion

This case report describes a rare occurrence of recurrent lipoma in the wrist of a 58-year-old female. The literature on such cases is limited, making it important to document and share this unique presentation. When encountering recurrent swelling with rapid growth, particularly in uncommon locations like the wrist, pre-operative ultrasound should be considered to facilitate appropriate planning. Pre-operative ultrasound should be considered an essential tool for facilitating appropriate planning. In complex cases, the use of imaging modalities, like an MRI, can significantly aid in surgical planning, especially in the case of potential anatomical anomalies [12]. Post-operative histopathological examination remains crucial for confirming the diagnosis and differentiating lipomas from other adipocytic tumors. Furthermore, this case report underscores the significance of meticulous surgical planning, scar management, and comprehensive postoperative rehabilitation for achieving optimal functional recovery. By emphasizing these aspects, healthcare professionals can contribute to the successful long-term outcomes of patients with recurrent lipoma or complex localization.
